# Navigating the Proteomic Landscape of Menopause: A Review

**DOI:** 10.3390/medicina60091473

**Published:** 2024-09-09

**Authors:** Basant E. Katamesh, Pragyat Futela, Ann Vincent, Bright Thilagar, Mary Whipple, Abdul Rhman Hassan, Mohamed Abuelazm, Sanjeev Nanda, Christopher Anstine, Abhinav Singla

**Affiliations:** 1 Division of General Internal Medicine, Mayo Clinic, Rochester, MN 55905, USA; el-fetouhkatamesh.basant@mayo.edu (B.E.K.); singla.abhinav@mayo.edu (A.S.); 2 Department of Internal Medicine, Metro Health Medical Center, Cleveland, OH 44109, USA; 3 School of Nursing, University of Minnesota, Minneapolis, MN 55455, USA; 4 Department of Ophthalmology, Wayne State University School of Medicine, Detroit, MI 48201, USA; 5 Faculty of Medicine, Tanta University, Tanta 31527, Egypt; dr.mabuelazm@gmail.com

**Keywords:** proteins, proteomics, biomarker, perimenopause, postmenopause, menopause

## Abstract

*Background and Objectives*: Proteomics encompasses the exploration of protein composition, regulation, function, and pathways. Its influence spans diverse clinical fields and holds promise in addressing various women’s health conditions, including cancers, osteoporosis, and cardiovascular disorders. However, no comprehensive summary of proteomics and menopausal health exists. Our objective was to summarize proteomic profiles associated with diseases and disorders in peri- and postmenopausal women. *Materials and Methods*: We conducted a comprehensive search of databases including PubMed, Google Scholar, the Cochrane database, Elsevier, and ScienceDirect until 2022. A total of 253 studies were identified, and 41 studies met the inclusion criteria to identify data of interest. These included the study design, disease, and proteomics/proteins of significance, as described by the authors. *Results*: The 41 studies covered diverse areas, including bone disorders (10 studies), cardiovascular diseases (5 studies), oncological malignancies (10 studies), and various conditions, such as obesity, nonalcoholic liver disease, the effects of hormone replacement therapy, and neurological diseases (16 studies). The results of our study indicate that proteomic profiles correlate with heart disease in peri- and postmenopausal women, with distinct sex differences. Furthermore, proteomic profiles significantly differ between women with and without osteoporosis. Additionally, patients with breast, ovarian, and endometrial cancer exhibit notable variations in proteomic profiles compared to those without these conditions. *Conclusions*: Proteomics has the potential to enhance risk assessment and disease monitoring in peri- and postmenopausal women. By analyzing unique protein profiles, clinicians can identify individuals with heightened susceptibility to specific diseases or those already affected by established conditions. This review suggests that there is sufficient preliminary data related to proteomics in peri- and postmenopausal women for early identification of cardiovascular disease, osteoporosis, and cancers, disease monitoring, and tailoring individualized therapies. Rigorous validation studies involving large populations are essential before drawing definitive conclusions regarding the clinical applicability of proteomic findings.

## 1. Introduction

Menopause, which occurs on average between the ages of 45 and 55, marks the end of a woman’s reproductive period. It is generally preceded by a perimenopausal phase characterized by ovarian hormonal fluctuations and irregular menstrual cycles [1]. Common symptoms during menopause include hot flashes, sleep disturbances, mood changes, and cognitive dysfunction [2,3]. Menopause can also be surgically induced in women who undergo bilateral salpingo-oophorectomy (BSO), which results in a sudden cessation of ovarian hormone production. Menopausal symptoms in these women tend to be more severe in comparison with natural menopause [2,4]. Medical conditions linked to menopause include osteoporosis, endometrial cancer, breast cancer, and cardiovascular diseases [5,6].

The decline in estrogen levels during the menopause transition is reported to increase the risk of cardiovascular disease and inflammation [7]. The menopause transition also affects various lipid parameters, including total cholesterol, low-density lipoprotein (LDL), high-density lipoprotein (HDL), and apolipoprotein, independent of aging [7]. Additionally, the prevalence of metabolic syndrome increases during the menopausal transition, also independent of aging [8,9,10,11]. The menopause transition period also adversely impacts measures of body composition and vascular health [7]. These changes contribute to a higher risk of plaque buildup, atherosclerosis, and cardiovascular disease reported in postmenopausal women [8]. Women who undergo surgical menopause are reported to have a higher risk of coronary artery diseases and stroke due to the rapid decline in estrogen, further strengthening this association [2,4]. Estrogen loss during the menopause transition also accelerates changes in bone density, heightening the risk of osteopenia and osteoporosis, a condition with significant morbidity in postmenopausal women [12]. As estrogen levels drop, bone resorption surpasses bone formation, leading to reduced bone mineral density and increased fracture susceptibility [12]. Women with menopause induced surgically are reported to have a higher risk of osteoporosis compared to women with natural menopause as a result of the rapid loss of estrogen [2,4]. Although the hormonal changes associated with the menopause transition are not directly linked to an increased risk of cancers, the prevalence of multiple cancers increases in the postmenopausal years, which is likely a function of age. The most common cancers reported in postmenopausal women are breast, lung, colon, endometrial, and ovarian cancers. The menopausal transition, which is associated with an increased risk of cardiovascular disease, osteoporosis, and cancers, presents a crucial window for early disease detection and treatment using novel and innovative techniques. Recent proteomics studies suggest that examining disease mechanisms at the proteome level could be instrumental in identifying biomarkers for conditions before they manifest [13,14]. Therefore, proteomic research offers an opportunity to identify biomarkers related to menopausal conditions prior to disease onset.

Protein activity, localization, and turnover are the primary processes that regulate physiological changes in the human body. Because the proteome is dynamic and it changes over time depending on physiological conditions, i.e., a healthy physiological state versus a pathological stage, the study of proteins has the potential for identifying disease-specific biomarkers and elucidating molecular mechanisms underlying disease states [13,15]. Proteomic studies involve collecting serum or plasma samples followed by processing proteins ex vivo and digesting them into peptides. Following this, separation techniques, including liquid chromatography (LC), Matrix-Assisted Laser Desorption/Ionization (MALDI), surface-enhanced laser desorption/ionization (SELDI), single-cell proteomics, and spectrometry-based glycoproteomics and glycomics assays, aid in protein identification [16,17,18]. The protein data generated from these techniques undergo computational analysis whereby proteins are matched against established proteomic databases [17,18]. Bioinformatics tools are employed to analyze and interpret the data in the context of biological pathways. Interpretation of proteomic data often involves integrating findings with existing biological knowledge and experimental validation [17,18]. Researchers correlate changes in protein expression or modification with known physiological functions or disease mechanisms to deduce pathways that are dysregulated in specific conditions. Peptide platforms are therefore powerful tools for clinical and biological inquiries, and they can guide individualized therapies, thus enhancing personalized medicine [19,20].

Proteomic studies related to menopause have shown promise in addressing menopausal-related conditions. By identifying and quantifying different proteins from biological samples using peptide platforms, researchers can compare the proteomes of premenopausal and postmenopausal women and determine the changes that occur and their physiological and pathophysiological implications [4]. For example, a study examined 71 biomarkers and identified seven proteins that were significantly associated with early menopause [21]. In another study, postmenopausal women with osteoporosis exhibited distinct proteomic profiles in comparison to postmenopausal women with osteopenia [22]. Proteomics has also been explored in breast, ovarian, and endometrial cancer, obesity, and cardiovascular disease in menopausal women. While these studies suggest a promising role for proteomics in menopause-related diseases, no comprehensive summary of proteomics and menopausal health exists to date. Our study aims to fill this gap by identifying relevant proteomic research and summarizing key findings and clinical implications.

## 2. Materials and Methods

### 2.1. Protocol and Search Strategy

A comprehensive search from 2010 to 28 October 2022 was conducted. The databases searched included Ovid MEDLINE(R) and Epub Ahead of Print, In-Process and Other Non-Indexed Citations, and Daily, Ovid EMBASE, the Ovid Cochrane Central Register of Controlled Trials, the Ovid Cochrane Database of Systematic Reviews, and Scopus. The search strategy was designed and conducted by an experienced research librarian with input from the study’s principal investigator. Controlled vocabulary supplemented with keywords was used to search for proteomic testing in menopausal women and animal models. MESH terms were added when applicable to retrieve relevant studies, including menopause, perimenopause, postmenopause, proteomics, glycoproteomic, etc.

### 2.2. Eligibility Criteria

We included original papers that discussed serum or plasma proteomics in peri- and postmenopausal women. The study types included case-control studies, cohorts, cross-sectional analysis, randomized clinical trials, non-randomized clinical trials, and diagnostic-test-accuracy assessments. Studies were excluded if (1) only tissue proteomics were studied, (2) the study was conducted on animals or cell culture, (3) the study population was restricted to include only men or premenopausal women, (4) the studies were not original papers (review articles, editorials, commentaries, etc.), or (5) the studies were published in languages other than English.

### 2.3. Screening and Data Extraction

All retrieved studies were imported into COVIDENCE, a standardized platform for systematic and narrative reviews [23]. Following this, duplicates were eliminated, and abstracts were screened for eligibility criteria. All studies that met the eligibility criteria were evaluated to identify data of interest. This included the study design, the disease studied, and proteomics/proteins of significance, as described by the authors.

## 3. Results

### 3.1. Study Identification, Screening, and Inclusion

**Figure 1:**
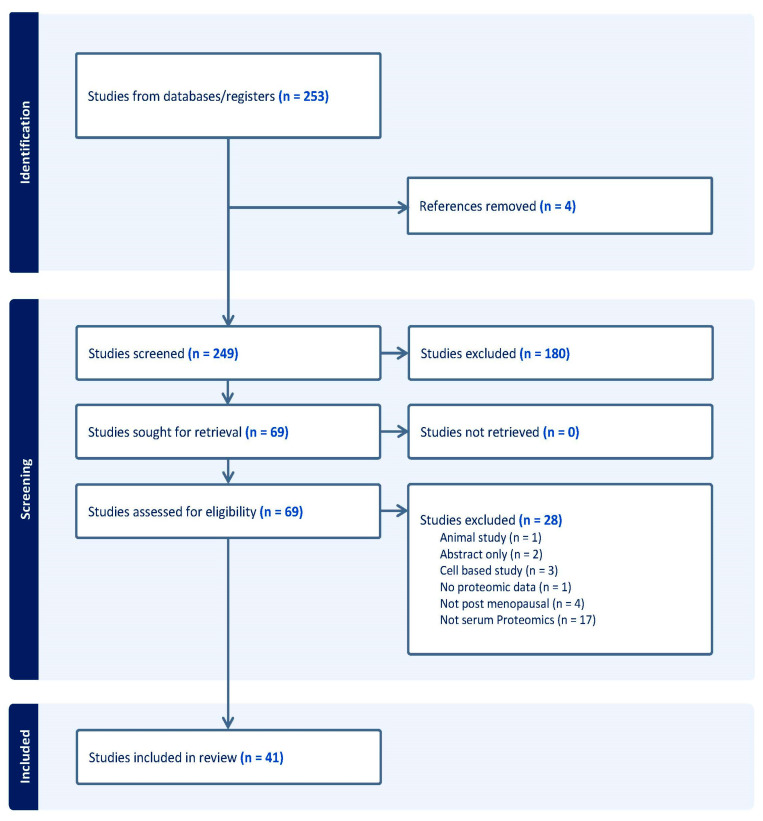
Study identification screening and inclusion.

Our searches identified 253 studies. After the removal of duplicates, 249 studies were eligible for abstract screening, and 69 underwent full-text screening. In total, 41 studies met the study criteria and were included in this review. Details of the screening process and the inclusion of studies are reported in Figure 1.

### 3.2. Characteristics of Included Studies

Of the 41 studies, most were case-control studies (23 studies), followed by cohort studies (12 studies), non-randomized clinical trials (3 studies), randomized clinical trials (2 studies), and one diagnostic test accuracy study [5,22,24,25,26,27,28,29,30,31,32,33,34,35,36,37,38,39,40,41,42,43,44,45,46,47,48,49,50,51,52,53,54,55,56,57,58,59,60,61,62]. The majority of these studies were conducted in the United States (21 studies), followed by Asia (7 studies) and Europe (5 studies). We included studies published until 2022, and the studies had a median (IQR) of 102 (26.5–388) participants. These studies explored a wide range of diseases and pathologies affecting the population. Specifically, five studies focused on proteomics, biomarkers, and pathways involved in cardiovascular health, ten focused on various cancers, and ten focused on bone diseases. The remaining 16 studies addressed topics including aging, migraine, weight loss, premature ovarian failure, and nonalcoholic fatty liver disease. The details of the included studies are presented in Table 1.

### 3.3. Proteomics in Cardiovascular Health

We identified five studies identifying over 50 protein markers and pathways associated with CVD. The most significant proteomic findings identified by these authors are reported in Table 1. Prentice et al. (2010, 2013) identified proteins, such as B2M, ORM1, and IGFALS, as potential CVD diagnostic markers for stroke and heart disease and B2M, IGFBP1, THBS1, and CFD as predictors of hyperlipidemia and hypertension [50,51]. Protein markers influencing cholesterol efflux capacity predicting cardiovascular disease were studied by Jin et al. (2019), who identified apolipoproteins AI, CI, CII, CIII, and CIV as potential biomarkers of CVD [38]. Lau et al. (2019) reported sex-based differences in the proteins associated with CVD. They identified apolipoprotein B, CD14, and pro-basic platelet protein as proteomic markers associated with an increased incidence of cardiovascular disease in women compared to men [41]. Similarly, Appiah et al. (2022) identified several proteins associated with atherosclerosis in peri- and postmenopausal women [5]. In summary, proteomics studies related to cardiovascular health and disease suggest that proteomics testing may potentially detect and predict coronary artery disease and stroke. Additionally, there may be gender differences in men and women with coronary artery disease.

### 3.4. Proteomics in Oncology

We identified 10 studies that reported proteomic biomarkers associated with the detection, diagnosis, and prognosis of various oncological diseases, including ovarian cancer, endometrial cancer, adnexal mass, breast lesions, and breast cancer. The most significant proteomic findings identified by these authors are reported in Table 1. Bergen et al. (2003) highlighted the association between Fibrinopeptide-A concentrations and their correlation with ovarian cancer stages [25]. Tarney et al. (2019) and Celsi et al. (2022) investigated differences in biomarkers in postmenopausal women with and without endometrial cancer and reported a combined difference of 71 proteins between the two groups [28,57]. Among these six proteomic markers, namely, complement factor B, serotransferrin, catalase, proteasome subunit beta type-6, beta-2-microglobulin, and protocadherin-18, they were reported to have high diagnostic potential for endometrial cancer, and suprabasin was identified as a potential marker for poor-prognostic endometrial cancer [28,57]. Watrowski et al. (2022) identified CA125 and osteopontin as proteins with high discriminating power and diagnostic index for adnexal mass malignancy [60]. Delmonico et al. (2016) reported differences in nine proteins in pre- and postmenopausal women with either fibroadenoma or invasive ductal carcinoma in comparison to controls [32]. Among the identified proteins, five were significantly altered in both disease states and included α-2-macroglobulin, ceruloplasmin, haptoglobin, hemopexin, and vitamin D binding protein [32]. Five studies investigated proteomics associated with breast cancer [27,39,43,45,48]. Katayama et al. (2019) examined prognostic proteins in triple-negative breast cancer and identified 43 proteins and three genes with prognostic value [39]. Piterri et al. (2010), Henderson et al. (2019), Li et al. (2011), and Carlsson et al. (2008) focused on breast cancer detection and collectively identified 125 significant proteins that can be implicated in early breast cancer detection, diagnosis, or its metastasis [27,43,45,48]. Among the identified proteins, epidermal growth factor receptor was found to be a significant predictor by Li et al. (2011) and Piterri et al. (2010) [43,48]. In summary, proteomic research may be potentially beneficial for the early detection of breast, ovarian, and endometrial cancer. It may also help predict cancer risk among hormone-therapy users and guide personalized management of these conditions.

### 3.5. Proteomics in Bone Health

We identified a total of 10 studies providing insight into the role of proteomics in osteoporosis and osteopenia. The most significant proteomic findings identified by these authors are reported in Table 1. Soh et al. (2022), Li et al. (2013), and Zhang et al. (2016) investigated osteoporosis in postmenopausal women, identifying 138 protein peaks, seven gene sets, and three proteins predictive of osteoporosis [44,55,62]. Daswani et al. (2015) studied osteoporosis in pre- and postmenopausal women, identifying 35 proteins significantly altered in postmenopausal women, including tHSP25 and pHSP25, which play a role in monocyte migration and osteoporosis development and therefore can be beneficial in disease prediction [31]. Shi et al. (2015) and Shi et al. (2017) investigated primary osteoporosis in postmenopausal women and reported that 16 protein peaks and 87 proteins were differentially expressed in postmenopausal women with and without osteoporosis [52,53]. Among these, GAS6, SPP24, RAB7A, and TSP are known to play a role in fibroblast proliferation, angiogenesis, and immune response and may offer insight into the mechanism of osteoporosis [52]. He et al. (2016) identified 10 protein peaks predictive of osteopenia in postmenopausal women; among the proteins identified, secretin was reported to have the highest predictive power [37]. Martinez-Aguilar et al. (2019), Huang et al. (2020), and Pepe et al. (2022) focused on postmenopausal women with either osteoporosis or osteopenia, identifying several proteins and miRNAs that can be utilized in predicting, diagnosing, and differentiating osteoporosis and osteopenia in postmenopausal women [22,46,47]. Among the identified proteins, ceruloplasmin, fibrinogen, vitronectin, clusterin, and apolipoprotein were reported to be most significant [46,47]. In summary, proteomics may be a potentially beneficial diagnostic tool for osteoporosis and osteopenia in pre- and postmenopausal women.

### 3.6. Proteomics in Other Disease States

The remaining 11 studies provided insight into the role of proteomics in understanding various diseases and conditions, such as obesity, migraine, cognitive impairment, and nonalcoholic fatty liver disease. The most significant proteomic findings identified by these authors are reported in Table 1. Four studies examined the impact of hormone replacement therapy (HRT); one study examining HRT’s impact on CVD, one studied HRT’s impact on breast cancer risk, and another studied the reproducibility of biomarkers over time [36,49,58,59]. Collectively, these studies identified over 200 proteomic biomarkers that had the potential to distinguish between HRT users and non-users [36,49,58,59]. Gericke et al. (2005) identified several proteomic markers, including differences in endothelin I/II and endothelin-converting enzymes, in cardiovascular disease between HRT users and non-users and highlighted their potential use to monitor inflammatory responses [36]. Thomas et al. (2022) identified 54 proteins altered in postmenopausal women with a history of HRT and their possible use to detect breast cancer in patients with prior hormone therapy [58]. The study also stated that proteomic changes associated with HRT were visible even years after stopping HRT [59]. This was also concluded by Tworoger et al. (2008), indicating that biomarkers can serve as long-term indicators of women’s health status [59]. Pitteri et al. (2009) compared proteomic differences between postmenopausal women on estrogen-only or combined HRT therapy [49]. They identified approximately 192 proteins that differed between the groups and concluded that proteomic differences can provide insight into the varying health outcomes associated with different hormone therapies [49].

Fuchs et al. (2007) investigated the effects of dietary intervention, i.e., soy isoflavones, on cardiovascular diseases [34]. They reported that 29 proteins, including Heat Shock Protein 70, lymphocyte-specific protein phosphatase, α-enolase, and galectin-1, had potential as biomarkers of atherosclerosis-preventive activities induced by soy isoflavone consumption [34]. Cai et al. (2018) explored proteomic changes associated with nonalcoholic fatty liver disease, identifying 167 proteins and highlighting serum RBP4 and LGAL3BP as potential early diagnostic markers of alcoholic liver disease in postmenopausal women [26]. Lal et al. (2019) examined proteomic markers related to cognitive impairment in postmenopausal women with obstructive sleep apnea [40]. They identified 22 proteins, including ceruloplasmin and adiponectin, that had potential for early disease prediction [40]. Lee et al. (2019) studied proteomics profiles in women with premature ovarian failure, identifying 11 proteins, including Ceruloplasmin, Complement C3, Fibrinogen α, Fibrinogen β, and Sex-Hormone-Binding Globulin (SHBG), that were predictive of premature ovarian failure [42]. Shin et al. (2022) investigated aging-related proteomic changes, revealing three proteins associated with chronological aging, namely Growth-Differentiation Factor 15, insulin-like growth factor binding protein-2, and tumor necrosis factor-a, and two proteins associated with menopausal age, namely IL-8 and monocyte chemoattractant protein-1, in addition to ten proteins associated with both chronological and menopausal age [54]. Bellei et al. (2020) investigated proteins associated with migraines in peri- and postmenopausal women [24]. Among the 12 proteins significantly associated with migraines, apolipoprotein A-I was the most significant in perimenopausal women, and transthyretin was most significant in postmenopausal women [24]. Wong et al. (2008) explored proteomic profiles associated with weight loss in postmenopausal women with a recent history of HRT use [61]. Among the 57 identified proteins, sustained weight loss was associated with decreased IL-1, IL-6, and C-reactive protein [61]. Similarly, JAK-STAT and NF-κB pathways were identified as significant obesity-associated pathways in another study by Garrison et al. (2017) [35].

Sun et al. (2019) also reported that 173 proteins were differently expressed between lean and obese postmenopausal women with increased breast density who were on Lovaza [56]. Among the proteins identified, gelsolin, vitamin D binding protein isoform 1 precursor, and fibronectin isoform 10 precursor were noted to be associated with favorable breast densities [56]. These findings have similarities to the study by Fabian et al., who reported favorable proteomic profiles related to breast cancer risk in postmenopausal patients supplemented with an omega-3-rich diet [33]. Finally, Dalenc et al. (2010) and Chao et al. (2013) identified proteins that could discriminate between responses to immune therapies and hormone therapies, respectively, towards breast cancer [29,30]. In summary, numerous studies suggest that proteomic research may have diverse future applications for personalized health management in postmenopausal women. These varied in the detection of conditions, such as nonalcoholic fatty liver disease, obstructive sleep apnea, cognitive impairment, migraine, and premature ovarian failure. Additionally, proteomic profiles may be beneficial for predicting treatment response to therapies, such as omega-3 fatty acids, Lovaza, soy isoflavones, and hormone therapy.

## 4. Discussion

As we enter a new era of clinical science, biomarkers play a crucial role in identifying the underlying causes of diseases and guiding targeted therapies. Traditionally, clinicians have relied on routine blood tests, such as cell counts, biochemical profiles, hormonal levels, inflammatory markers, and lipid levels, to evaluate patients’ health. There has been an evolution of lab testing to the point that more than 7000 proteins can be tested quickly in a single sample, yet the interplay between these proteins and the occurrence of disease is an area we are just beginning to understand. This review elucidates the effectiveness of proteomic approaches in understanding, diagnosing, and predicting outcomes across diverse pathologies affecting the postmenopausal population, including osteoporosis, cancers (breast, ovarian, and endometrial), and cardiovascular diseases (stroke, coronary heart disease, and atherosclerosis), among others.

The results of our review demonstrate that proteomics could play a pivotal role in the future in advancing disease screening and the early detection of various conditions in asymptomatic patients. Multiple studies in this review support the role of proteomics in the early detection of coronary heart disease, breast, ovarian, and endometrial cancer, cognitive impairments, and premature ovarian failure [25,32,40,42,43,50,51,57]. For example, Bergen et al. (2003) demonstrated the role of Fibrinopeptide-A in the early detection of ovarian cancer, Li et al. (2011) demonstrated the role of EGFR in the early detection of early breast cancer, and Tarney et al. (2019) demonstrated the diagnostic potential of a 47-protein panel in the early diagnosis of endometrial cancer [25,43,57].

Cardiovascular disease, which is currently the leading cause of death in women, also contributes substantially to the health disparity between men and women in the United States [63]. Prentice et al. (2010, 2013) and Jin et al. (2019) demonstrated the diagnostic potential of proteomic profiles in the detection of cardiovascular disease in women [38,50,51]. Future strategies, such as validated predictive cardiovascular proteomics platforms, offer promising avenues for the early detection and prevention of cardiovascular disease in women, thus aiming to narrow the current health gap and improve long-term outcomes. Lau et al. (2019) suggested that the mechanisms of cardiovascular disease may be different in women and men. In their study, they noted significant differences in predictive proteins, such as apolipoprotein B, adipokines, and inflammatory markers, between men and women [41]. This highlights the need for sex-specific proteomic studies specific to cardiovascular health [41].

Breast cancer, the most common malignancy among women worldwide, remains a formidable health challenge [64]. It affects millions of women annually and ranks as one of the leading causes of cancer-related deaths. Beyond the physical toll, breast cancer imposes a substantial economic burden. Healthcare costs associated with diagnosis, treatment, and follow-up care contribute significantly to this impact. Additionally, lost productivity due to illness affects both patients and their families, further straining the economy. Ovarian and endometrial cancer, although less prevalent than breast cancer, also carry a significant mortality risk. Early detection remains a challenge, and treatment costs add to the economic burden. Currently, only breast cancer benefits from a clinically available screening tool—mammography. However, this method has limitations, including false positives and patient discomfort. For ovarian and endometrial cancers, no established screening methods exist. In this review, case-control studies by Piterii et al. (2010) and Li et al. (2011), and a cohort study by Henderson et al. (2019), demonstrated the potential utility of proteomics for breast cancer detection [43,45,48]. Similarly, case-control studies by Bergen et al. (2003) and Tarney et al. (2019) demonstrate the potential utility of proteomic biomarkers in the early detection of ovarian and endometrial cancer [25,57]. These approaches deserve further research and validation. Serum biomarkers specific to these cancers could revolutionize early detection by enabling timely intervention and improving outcomes.

Proteomic biomarkers could facilitate the monitoring of therapeutic efficacy and personalized interventions, thus guiding treatment optimization and individualized care. For instance, Sun et al. (2019) reported differential effects on proteomic profiles in obese and non-obese women, suggesting that proteomic biomarkers can facilitate the monitoring of therapeutic efficacy [56]. Additionally, Dalenc et al. (2010) reported that fibrinogen alpha could discriminate between responders and non-responders in a cohort of women with resistant metastatic breast cancer [30]. Similarly, Gericke et al. (2005) reported the differential influence of hormone therapy on inflammatory response associated with cardiovascular disease [36]. These insights underscore the importance of proteomics in tailoring treatment and improving patient outcomes.

Proteomic studies could also offer beneficial insight into underlying disease mechanisms and pathways, shedding light on disease pathogenesis and progression. For instance, Huang et al. (2020) found that proteins, such as Lysozyme C, glucosidase, and protein disulfide isomerase A5, were differentially associated with bone density [22]. Additionally, Shi et al. (2017) reported differential expression of proteins involved in fibroblast proliferation, angiogenesis, and immune response in postmenopausal women with osteoporosis [52]. Further study of these proteins and associated pathways could help us better understand the disease mechanism underlying osteoporosis. Similarly, Garrison et al. (2017) explored the role of specific pathways in obesity, identifying JAK-STAT and NF-κB pathways as important regulators [35]. Further study of these pathways could help us better understand the pathophysiology of obesity and develop personalized interventions.

## 5. Conclusions

Proteomics has the potential to facilitate early identification of diseases and advance our understanding of disease mechanisms, thus ultimately improving patient outcomes. This review suggests that there are sufficient preliminary data to recommend future research into proteomics for peri- and postmenopausal women across a variety of disease conditions. Rigorous validation studies involving large populations are essential before drawing definitive conclusions about the clinical applicability of proteomic findings.

## 6. Future Directions

The future of proteomics in women’s health looks promising in many regards. These include the development of targeted biomarker panels for early disease detection, leveraging proteomics to tailor targeted individualized treatments, optimizing drug response, and developing a deeper understanding of biological dysregulations that underlie important health concerns in women’s health.

## Figures and Tables

**Table 1:** Summary of proteomic studies.

Author, Year, Country	Disease Studied	- Study Design - Sample Size (n) - Sample Tested (i.e., Blood, Plasma, Serum)	Inclusion Criteria	Significant Findings Reported by Study Authors	Narrative Synthesis
Prentice 2010 [50] United States	Stroke and CHD	- Case-control - n = 1600 - Blood	Postmenopausal women with CHD or stroke	Identified 37 significant proteins (*p* < 0.05) for CHD. Beta-2 microglobulin (B2M), alpha-1-acid glycoprotein 1 (ORM1), and insulin-like growth factor binding protein acid labile subunit (IGFALS) had a false discovery rate < 0.05. Identified 47 significant proteins (*p* < 0.05) for stroke. Apolipoprotein A-II precursor (APOA2), peptidyl-prolyl isomerase A (PPIA), and insulin-like growth factor binding protein 4 (IGFBP4) had a false discovery rate < 0.05.	Proteomics may be potentially beneficial for the detection of CHD and stroke in postmenopausal women.
Prentice 2013 [51] United States		- Case-control - n = 710 - Blood		Identified 3 significant proteins (*p* < 0.05) for CHD: beta-2 microglobulin (B2M), thrombospondin-1 (THBS1), and complement factor D pre-protein (CFD). A one-year increase in insulin-like growth factor binding protein 1 (IGFBP1) can suggest CHD risk in women initiating oral hormone therapy. Identified 3 significant proteins (*p* < 0.05) for stroke: beta-2 microglobulin (B2M), insulin-like growth factor binding protein 2 (IGFBP2), and insulin-like growth factor binding protein 4 (IGFBP4).	
Jin 2019 [38] UK, Canada, and United States	CEC	- Case-control - n = 137 - Serum	Postmenopausal women with a history of CAD	Identified five apolipoproteins (Apo AI, CI, CII, CIII, and CIV) as estimators of predicted CEC and cardiovascular disease in postmenopausal women with a history of CAD.	Proteomics may be potentially beneficial for the prediction of CAD in postmenopausal women.
Lau 2019 [41] United States	CVD	- Cohort - n = 7184 - Blood	Men and women with no prevalent CVD at the time of enrollment	Identified several biomarkers differentially associated with incident CVD events in women compared to men, including apolipoprotein B, CD14, and pro-basic platelet protein (PPBP). Found that adipokines and inflammatory markers, such as leptin and C-reactive protein, were higher in women than men.	Proteomics may be potentially beneficial as predictive disease markers for CVD in women and men. Proteins and pathways associated with CVD are differentially expressed in men and women.
Appiah 2022 [5] United States	Atherosclerosis	- Cohort - n = 4508 - Blood	Peri- and postmenopausal women aged 45–64 years	Identified 38 proteins that were significantly altered between pre- and postmenopausal women at baseline. Identified 29 proteins that significantly changed between the pre- and postmenopausal groups during follow-up, with notable changes in putative hydrolase retinoblastoma-binding protein 9 (RBBP9), collagen αlpha 2(XI) chain, chondroadherin, ferritin light chain, guanylate-binding protein 1, R-spondin-1, secreted frizzled-related protein 4, WD repeat-containing protein 5, LH, and FSH.	Proteomics may be potentially beneficial for the prediction of CAD in peri- and postmenopausal women.
Bergen 2003 [25] United States	Ovarian cancer	- Case-control - n = 18 - Serum	Women with stage III/IV ovarian cancer or age-matched controls	Identified over 15 molecular entities that differed between ovarian cancer patients and controls. Found that Fibrinopeptide-A concentrations were significantly correlated with ovarian cancer stage and notably higher in patients compared to controls.	Proteomics may be potentially beneficial for the early detection of ovarian cancer.
Tarney 2019 [57] United States	Endometrial cancer	- Case-control - n = 224 - Blood	Postmenopausal women aged 55–74 years with and without endometrial cancer	Identified 47 proteins that were significantly altered between cases and controls diagnosed with endometrial cancer. Found that complement factor B, serotransferrin, catalase, proteasome subunit beta type-6, beta-2-microglobulin, and protocadherin-18 exhibited high diagnostic potential.	Proteomics may be potentially beneficial as a diagnostic study of endometrial cancer in postmenopausal women.
Celsi 2022 [28] Italy		- Cohort - n = 103 - Serum	Postmenopausal women with endometrial cancer and controls	Identified 24 biomarkers that differed between postmenopausal women with and without endometrial cancer. Identified suprabasin as a potential marker for poor prognosis in endometrial cancer, with Isoform 1 upregulated and Isoform 2 downregulated.	Proteomics may be potentially beneficial to predict the risk of endometrial cancer in postmenopausal women.
Watrowski 2022 [60] Germany, Austria, and Belgium	Adnexal mass	- Cohort - n = 77 - Plasma	Pre- and postmenopausal women with benign or malignant ovarian tumors	Identified cancer antigen 125 (CA125) and osteopontin as having the best discriminating power as single markers of adnexal mass. CA125, osteopontin, prolactin, macrophage migration inhibitory factor, and, optionally, Human Epididymis Protein 4 (HE4) and leptin, when combined, are promising for diagnosing adnexal mass.	Proteomics may be potentially beneficial to predict the risk of malignancy in adnexal masses in pre- and postmenopausal women.
Delmonico 2016 [32] Brazil	Impalpable breast lesions (fibroadenoma and infiltrative ductal carcinoma)	- Case-control - n = 28 - Blood and plasma	Pre- and postmenopausal women with impalpable breast lesions BI-RADS grade 3 or 4 and healthy controls	Identified 6 proteins significantly altered in fibroadenoma and 3 proteins significantly altered in infiltrative ductal carcinoma. Found that αlpha 2-macroglobulin, ceruloplasmin, haptoglobin, hemopexin, and vitamin D binding protein were the most significantly altered proteins in both diseases.	Proteomics may be potentially beneficial for the diagnosis of fibroadenomas and infiltrative ductal carcinomas in pre- and postmenopausal women.
Katayama 2019 [39] United States	Triple-negative breast cancer	- Cohort - n = 48 - Blood	Pre- and postmenopausal women with triple-negative breast cancer (stage II and III)	Identified 43 proteins associated with tumor regression in triple-negative breast cancer. Determined that the genes chloride intracellular channel 1 (CLIC1), limbic system-associated membrane protein (LSAMP), and microtubule-associated protein RP/EB family member 1 (MAPRE1) were significant regardless of tumor stage, grade, size, or menopausal status.	Genes may be potentially beneficial prognostic indicators for triple-negative breast cancer in pre- and postmenopausal women.
Pitteri 2010 [48] United States	Breast cancer amongst hormone-therapy users	- Case-control - n = 490 patients - n = 490 controls - Blood	Estrogen-receptor-positive (ER+) breast cancer patients and matched non-cancer patients	Identified 57 proteins with significant changes (*p* < 0.05) between ER+ breast cancer patients and matched non-cancer controls. Identified epidermal growth factor receptor (EGFR) as a potential predictor of ER+ breast cancer.	Proteomics may be potentially beneficial in predicting cancer amongst hormone-therapy users.
Henderson 2019 [45] United States	Breast cancer (invasive breast cancer and ductal carcinoma in situ)	- Cohort - n = 469 in the training set - n = 194 in the validation set - Blood	Postmenopausal women aged ≥50 years with a BI-RADS category of 3, 4, or 5	Researched and validated a combinational proteomic biomarker assay for women aged ≥50 years, demonstrating a negative predictive value of 98% and a sensitivity of 93% when combined with clinical data.	Proteomics may be potentially beneficial for the diagnosis of breast cancer in postmenopausal women with invasive breast cancer or ductal carcinoma in situ.
Li 2011 [43] United States	Breast cancer	- Case-control - n = 396 validation participants n = 980 discovery - Blood	Breast cancer and matched healthy individuals	Identified 57 proteins that differentiate between cancer patients and controls. Found that epidermal growth factor receptor (EGFR) was elevated in breast cancer patients.	Proteomics may be potentially beneficial for early detection of breast cancer.
Carlsson 2008 [27] Sweden	Metastatic breast cancer	- Case-control - n = 40 - Blood	Postmenopausal women with metastatic breast cancer and healthy matched controls	Identified 11 biomarker signatures with significant changes between metastatic breast cancer patients and healthy controls, comprising 9 non-redundant proteins. Found that 5 proteins were upregulated (sialyl Lewisx, C3, C4, C5, and IL-8) while 4 were downregulated (transmembrane peptide, IL-5, IL-7, and MCP-3) in cancer patients.	
Soh 2022 [55] Malaysia	Osteoporosis	- Case-control - n = 40 - Blood	Postmenopausal women with and without osteoporosis	Identified platelet-derived growth factor-BB, IL-6 receptor, and tissue inhibitor of metallopeptidase-2 as differentiating between postmenopausal women with osteoporosis and those without. Found that IL-6 receptor was significantly lower in postmenopausal women with osteoporosis than those with normal bone health after adjusting for body mass index.	Proteomics may be potentially beneficial as a diagnostic tool for osteoporosis in postmenopausal women.
Li 2013 [44] China		- Case-control - n = 180 - Blood	Postmenopausal women aged 50–68 years with and without osteoporosis	Identified 138 different protein peaks, with 3167.4, 4071.1, 7771.7, and 8140.5 m/z showing the highest discriminatory power between postmenopausal women with and without osteoporosis.	
Zhang 2016 [62] United States		- Case-control - n = 42 - Blood	Postmenopausal women with extremely low or high bone mass density (BMD)	Identified four genes—Peptidylprolyl isomerase A, similar to Peptidylprolyl isomerase A isoform 1, Transgelin 2, and Isoform Long of 14-3-3 protein beta/alpha—that were significantly downregulated in postmenopausal women with extremely low BMD. Found that three genes—Lamin B1, Annexin A2-like protein, and Annexin A—were significantly upregulated in postmenopausal women with extremely low BMD.	
Daswani 2015 [31] India		- Case-control - n = 200 - Blood	Premenopausal women aged 30–40 years and postmenopausal women aged 50–60 years with and without osteoporosis	Identified 45 proteins significantly differentiating low from high bone mass density. Found that Heat Shock Protein 27 (HSP27) and phosphorylated Heat Shock Protein 27 (pHSP27) were significantly upregulated in low BMD in both pre- and postmenopausal women.	Proteomics may be potentially beneficial as a diagnostic tool for osteoporosis and osteopenia in pre- and postmenopausal women.
Shi 2015 [53] China	Primary type I osteoporosis	- Diagnostic test accuracy study - n = 25 - Blood	Postmenopausal women aged 50–70 years with and without osteoporosis	Identified 16 peaks differentiating postmenopausal women with osteoporosis from those without, with 8909.047, 8690.658, 13,745.48, and 15,114.52 m/z being significant.	Proteomics may be potentially beneficial as a diagnostic tool for osteoporosis in postmenopausal women.
Shi 2017 China [52]	Primary osteoporosis	- Case-control - n = 20 - Blood	Postmenopausal women with and without osteoporosis	Identified 87 proteins significantly differentiating postmenopausal women with osteoporosis from controls. Found that Ras-related protein Rab-7a (RAB7A), Thrombospondin-1 (TSP1), Growth arrest-specific protein 6 (GAS6), and Secreted phosphoprotein 24 (SPP24) were upregulated in postmenopausal osteoporosis.	
He 2016 [37] China	Osteopenia	- Case-control - n = 20 - Blood	Postmenopausal women with and without osteopenia	Identified 10 different peaks differentiating postmenopausal women with osteopenia from those without, from which peaks of 1699 and 3038 (Secretin) Da were the most significant.	Proteomics may be a potentially beneficial as a diagnostic tool for osteopenia in postmenopausal women.
Martinez-Aguilar 2019 [46] Mexico	Low bone mineral density	- Case-control - n = 30 - Blood	Postmenopausal women with normal BMD, osteoporosis, or osteopenia	Identified 27 proteins significantly differentiating low from high bone mass density. Found that low serum vitamin D-binding protein (VDBP) levels correlate with low BMD (osteopenic and osteoporotic) and osteoporotic fractures. Determined that ceruloplasmin showed significance in osteoporosis.	Proteomics may be potentially beneficial as a diagnostic tool for osteoporosis and osteopenia in postmenopausal women.
Huang 2020 [22] China	Osteoporosis and osteopenia	- Case-control - n = 54 - Plasma	Postmenopausal women, 18 with primary osteoporosis, 18 with osteopenia, and 18 with normal bone mass	Identified 8 significant proteins in osteopenia, 127 in osteoporosis, and 3 distinguishing osteoporosis from osteopenia. Found that Lysozyme C was negatively associated with BMD, while glucosidase and protein disulfide isomerase A5 were positively associated with BMD values.	
Pepe 2022 [47] Italy		- Case-control - n = 102 - Blood	Postmenopausal women aged ≥45 years with normal BMD, osteoporosis, or osteopenia	Identified patterns in extracellular vesicle (EV) content from patients with varying bone mass, with 63 miRNAs expressed; miR-1246 and miR-1224-5p were regulated in the osteoporotic group. Found decreased levels of fibrinogen, vitronectin, and clusterin and increased levels of coagulation factors and apolipoprotein in the osteoporotic group.	
Gericke 2005 [36] Germany	Hormone replacement therapy in cardiovascular disease	- Case-control - n = 40 - Serum	Postmenopausal women aged >45 years	Identified endothelin I/II and endothelin-converting enzyme as marker proteins with differing concentrations between hormone-therapy users and non-users. Found a significant difference in intensity for a protein with a molecular weight of 20,787 Da between hormone-therapy users and non-users. Reported that many of the identified markers are key participants in inflammation and cardiovascular disease development, including haptoglobulin, haptoglobulin-1-chain, apolipoprotein A, IL-6, tumor necrosis factor, endothelin I/II, endothelin-converting enzyme, and acute-phase reactants, such as C-reactive protein (CRP).	Proteomics may be potentially beneficial in evaluating inflammatory responses associated with cardiovascular disease in postmenopausal women on hormone therapy.
Thomas 2022 [58] Sweden	Hormone therapy and breast cancer risk	- Case-control - n = 549 - Plasma	Pre- and postmenopausal women, breast cancer patients, and age-matched controls	Identified a unique and persistent proteomic signature, including 54 significant proteins (*p* < 0.05), associated with prior hormone-replacement-therapy use. Among the 25 most significant proteins, 16 were upregulated and 15 were downregulated in patients more likely to have used hormone therapy.	Proteomics may be potentially beneficial in predicting the risk of breast cancer in pre- and postmenopausal women with a history of prior hormone therapy.
Tworoger 2008 [59] United States	Reproducibility of proteomics in non-hormone users	- Cohort - n = 60 - Blood	Postmenopausal women not taking HRT over 3 years	Identified protein peaks that were reproducible over time, suggesting that a single sample is sufficient.	Proteomic profiles are reproducible longitudinally.
Pitteri 2009 [49] United States	Hormone replacement therapy (estrogen vs. estrogen-plus-progestin)	- Cohort - n = 100 - Blood	Healthy postmenopausal women aged 50–79 years with a minimum of a 3-month hormone therapy washout period	Identified 98 proteins exhibiting a false discovery rate < 0.05 associated with changes under estrogen plus progestin therapy compared to 94 proteins for estrogen alone.	Proteomics profiles differ based on the type of hormone therapy, i.e., estrogen versus estrogen plus progesterone.
Fuchs 2007 [34] United Kingdom	Soy isoflavones intervention in cardiovascular diseases	- Randomized clinical trial - n = 10 - Blood	Postmenopausal women aged 45–70 years	Identified 29 proteins significantly different between postmenopausal women consuming soy isoflavones and those not consuming soy isoflavones. Found an increase in Heat Shock Protein 70, lymphocyte-specific protein phosphatase, and alpha-enolase, while galectin-1 decreased after soy extract consumption.	Proteomic profiles of postmenopausal women who consumed soy isoflavones were observed to be different from those who did not.
Cai 2018 [26] China	Nonalcoholic fatty liver disease	- Cohort - n = 153 - Blood	Pre- and postmenopausal women aged 34–56 years diagnosed with nonalcoholic fatty liver disease (NAFLD)	Identified 167 proteins significantly differentiating women with NAFLD from controls, with 65 upregulated and 102 downregulated in NAFLD. Determined that Retinol Binding Protein 4 (RBP4), galectin-3 binding protein (LGALS3BP), Histidine-rich Glycoprotein, and Peroxiredoxin-6 were associated with liver steatosis in postmenopausal women.	Proteomics may be potentially beneficial as a diagnostic tool for the detection of nonalcoholic fatty liver disease in pre- and postmenopausal women.
Lal 2019 [40] United States	Cognitive impairment in obstructive sleep apnea	- Case-control - n = 12 - Blood	Postmenopausal women aged 45–60 years with obstructive sleep apnea with or without cognitive impairment	Identified 22 proteins significantly up- or downregulated in obstructive sleep apnea. Found that increased insulin, prostasin, angiopoietin-1, plasminogen activator inhibitor 1, and interleukin-1 beta were associated with obstructive sleep apnea with cognitive impairment. Observed under-expression of Cathepsin B, ceruloplasmin, and adiponectin in cognitive impairment.	Proteomics may be potentially beneficial as a diagnostic tool for the detection of obstructive sleep apnea and associated cognitive impairment in postmenopausal women.
Lee 2019 [42] Korea	Premature ovarian failure	- Case-control - n = 171 - Blood	Women diagnosed with premature ovarian failure, those at risk, and healthy controls	Identified 11 proteins significantly differentiating cases of premature ovarian failure from controls. Found that Ceruloplasmin, Complement C3, Fibrinogen α, Fibrinogen β, and Sex-Hormone-Binding Globulin (SHBG) increased in patients with premature ovarian failure compared to controls.	Proteomics may be potentially beneficial as a diagnostic tool for the detection of premature ovarian failure.
Shin 2022 [54] Korea	Aging	- Cohort - n = 76 - Plasma	Postmenopausal women aged 46–82 years	Identified Growth-Differentiation Factor 15 (GDF15), insulin-like growth factor binding protein-2 (IGFBP-2), and tumor necrosis factor-alpha (TNF-alpha) as positively associated with chronological age. Found that IL-8 and monocyte chemoattractant protein-1 were associated with menopausal age and years since menopause. Identified 10 proteins associated with both chronological age and menopausal state: GDF15, interferon-gamma, IGFBP-2, IGFBP-7, IL-15, IL-1beta, IL-17A, IL-8, MCP-1, tissue inhibitors of metalloproteinase-2 (TIMP-2), TNF-alpha, vascular endothelial growth factor-A (VEGF-A), and interferon-inducible protein 10 (IP-10).	Proteomic profiles may provide insight into how menopause relates to proteomic indicators of aging.
Bellei 2020 [24] Italy	Migraine	- Non-randomized clinical trial - n = 45 - Serum	Peri- and postmenopausal women with primary or secondary headaches	Identified 12 proteins correlating with migraine patients. Found that Prothrombin, serum amyloid P-component, Ig kappa chain C region, apolipoprotein A-I, and serum amyloid A-4 protein were present in both menstrual-related migraine and migraine in postmenopause groups. Observed significant upregulation of 2 proteins and downregulation of apolipoprotein A-I (most significant) in perimenopausal women with headaches. Detected significant dysregulation of 4 proteins (tetranectin, alpha-1 antitrypsin, haptoglobin, and apolipoprotein A-IV) in postmenopausal women with headaches, with transthyretin being the most significant.	Proteomics may be potentially beneficial as a diagnostic tool for migraines in peri- and postmenopausal women.
Wong 2008 [61] United States	Weight loss	- Cohort - n = 290 - Blood	Postmenopausal women aged 52–62 years with a recent history of HRT use	Identified 57 cytokines differing between baseline and follow-up. Found that sustained weight loss was associated with a decrease in levels of IL-1 receptor antagonist, IL-6, and C-reactive protein.	Proteomic profiles associated with weight loss were observed to demonstrate biological variability over time.
Garrison 2017 [35] United States	Obesity	- Cohort - n = 924 - Plasma	Postmenopausal women with normal (<24.5 kg/m^2^) or high BMI (>25.0 kg/m^2^)	Identified JAK-STAT and NF-κB pathways as important regulators of obesity.	Proteomics may be potentially beneficial as a diagnostic tool for obesity in postmenopausal women.
Sun 2019 [56] United States	Effect of Lovaza (n-3FA) on breast density in obese and non-obese women	- Randomized clinical trial - n = 10 - Plasma	Lean (BMI ≤ 25) and obese (BMI ≥ 30) postmenopausal women with a breast density ≥25%	Detected 173 proteins differentially expressed between lean and obese postmenopausal women. Found that hemopexin precursor, vitamin D binding protein isoform 1 precursor, fibronectin isoform 10 precursor, and α-2 macroglobulin precursor were altered in a tumor-protective manner by an omega-3-rich diet in obese women. Identified gelsolin, vitamin D binding protein isoform 1 precursor, and fibronectin isoform 10 precursor as protective against breast densities and reduced by an omega-3-rich diet.	Proteomic profiles of lean and obese postmenopausal women who consumed Lovaza were observed to be different from those who did not.
Fabian 2015 [33] United States	High-dose omega-3 fatty acids and breast cancer risk	- Non-randomized clinical trial - n = 34 - Blood	Postmenopausal women <65 years old with increased risk of breast cancer	Observed favorable modulation in serum adiponectin, TNF-alpha, HOMA 2B (pancreatic beta cell function), and bioavailable estradiol, potentially reducing the risk of estrogen-driven breast cancer.	Proteomic profiles of postmenopausal women with increased risk of breast cancer who consumed high-dose omega-3 fatty acids were observed to be different from those who did not.
Dalenc 2010 [30] France	Tipifarnib plus Tamoxifen in Tamoxifen-resistant metastatic breast cancer	- Non-randomized clinical trial - n = 20 - Blood	Postmenopausal women with at least one measurable breast lesion	Found that fibrinogen α could discriminate between Tipifarnib and Tamoxifen treatment responders and non-responders. Identified p5900, a degradation product, as being potentially associated with invasion and/or inflammation.	Proteomics could potentially distinguish between responders and non-responders to the combination of Tamoxifen and Tipifarnib in postmenopausal women with metastatic breast cancer resistant to Tamoxifen.
Chao 2013 [29] United States	Impact of hormone therapy on the immune response towards breast cancer antigens	- Case-control - n = 380 - Plasma	Postmenopausal women with ER+ breast cancer and healthy controls	Identified multiple biomarkers associated with an immune response to breast cancer antigens. Found that IL-6 and other cytokines were reduced in hormone-therapy users.	Proteomic profiles may be potentially beneficial in the detection of immune response to breast cancer antigens in postmenopausal women with ER+ breast cancer.

CHD: coronary heart disease; CEC: cholesterol efflux capacity; CAD: coronary artery disease; CVD: coronary vascular disease; CD14: Cluster of Differentiation 14; WD: repeat-containing protein 5; LH: luteinizing hormone; FSH: follicle-stimulating hormone; BI-RADS: Breast Imaging Reporting and Data System; C3: Complement 3; C4: Complement 4; C5: Complement 5; IL: interleukin; MCP: monocyte chemoattractant protein; m/z: Mass-to-Charge Ratio; BMI: body mass index.

## Data Availability

Data are available upon request from the corresponding author.
